# Intermittent Light Exposures in Humans: A Case for Dual Entrainment in the Treatment of Alzheimer's Disease

**DOI:** 10.3389/fneur.2021.625698

**Published:** 2021-03-09

**Authors:** Mariana G. Figueiro, Sagan Leggett

**Affiliations:** ^1^Department of Population Health Science and Policy, Icahn School of Medicine at Mount Sinai, New York, NY, United States; ^2^Lighting Research Center, Rensselaer Polytechnic Institute, Troy, NY, United States; ^3^Department of Biological Sciences, Rensselaer Polytechnic Institute, Troy, NY, United States

**Keywords:** Alzheimer's disease, circadian entrainment, flashing light, gamma entrainment, memory, sleep

## Abstract

Circadian sleep disorders are common among American adults and can become especially acute among older adults, especially those living with Alzheimer's disease (AD) and mild cognitive impairment (MCI), leading to the exacerbation of symptoms and contributing to the development and advancement of the diseases. This review explores the connections between circadian sleep disorders, cognition, and neurodegenerative disease, offering insights on rapidly developing therapeutic interventions employing intermittent light stimuli for improving sleep and cognition in persons with AD and MCI. Light therapy has the potential to affect sleep and cognition via at least two pathways: (1) a regular and robust light-dark pattern reaching the retina that promotes circadian phase shifting, which can promote entrainment and (2) 40 Hz flickering light that promotes gamma-wave entrainment. While this is a new area of research, preliminary evidence shows the potential of dual circadian and gamma-wave entrainment as an important therapy not only for those with AD, but for others with cognitive impairment.

## Introduction

Forty-five percent of Americans report sleep problems that affect their daily activities at least once per week, with 35% reporting poor or fair sleep quality and 20% reporting that they did not feel refreshed by sleep on any day of the past week (1, 2). Asynchrony between normal work and social schedules and the timing of the internal clock (3) can lead to sleep disorders and sleep deprivation, particularly if the asynchrony is prolonged for an extended period, which in turn can negatively affect task performance, cognition, and general health (4–6). Sleep disorders and their attendant decrements can become especially acute among older adults, especially those living with Alzheimer's disease (AD) and mild cognitive impairment (MCI), leading to the exacerbation of symptoms and contributing to the development and advancement of the diseases (7–9). In fact, of the estimated 5.8 million people in the United States living with AD and related dementias (ADRD) (10), at least one-third experience difficulty sleeping (11, 12) and approximately two-thirds of their estimated 18.5 million unpaid caregivers report sleep disturbances themselves (10, 13, 14).

This review explores the connections between circadian sleep disorders, cognition, and neurodegenerative disease, offering insights on rapidly developing therapeutic interventions employing entraining light stimuli (both continuous and intermittent) for the treatment of sleep disorders, including in those with AD and MCI. Light therapy has the potential to affect sleep and cognition via at least two pathways: (1) a regular and robust light-dark pattern reaching the retina that promotes circadian phase shifting and thus, entrainment and (2) 40 Hz flickering light that promotes gamma wave entrainment. Both are discussed below.

## Circadian Rhythms

Circadian rhythms are endogenously driven biological rhythms that have a period close to 24 h and that can be entrained by exogenous time cues. Circadian rhythms are generated and regulated by a biological clock located in the suprachiasmatic nuclei (SCN) in the hypothalamus in the brain. In the absence of external cues, these rhythms free-run with a period close to, but not exactly 24 h (15). In humans, circadian rhythms free-run with an average period of 24.2 h. Light-dark patterns reaching the retina are the major synchronizers of circadian rhythms to the local position on earth and to the 24-h solar day, a process referred to as entrainment (16). Given that the circadian system in humans free-run with a period slightly longer than 24 h, the human circadian system needs to receive light after minimum core body temperature (usually in the morning hours) to maintain daily entrainment. This is because morning light will advance the timing of the clock while evening light (prior to minimum core body temperature) will delay the timing of the clock. It should be noted, however, that in general, entrainment is assessed in controlled laboratory conditions, while the studies performed in the field measure phase shifting, such as phase advance or phase delay using downstream outcome measures, such as sleep-wake cycle. As befits a diurnal species, the human biological clock interacts with the sleep-wake cycle to maintain waking during the day and sleep at night. The sleep-wake cycle is regulated by two systems, the circadian system and the homeostatic system (17, 18). Sleep consolidation and quality are reported to be best when the circadian and homeostatic systems are aligned (19). With greater time awake, homeostatic mechanisms increase sleep pressure as bedtime approaches. The circadian system sends an alerting signal to the body to counteract sleep pressure during the day and a sleeping signal during the night, promoting a consolidated night of sleep.

## Sleep, Brain Activity, and Brain Pathology

Studies have shown that non-rapid eye movement (NREM) sleep, rapid-eye movement (REM) sleep, and slow oscillations (SOs, 0.3–1 Hz, detected in the cerebral cortex during NREM sleep, when neuronal activity is synchronized) are associated with improvement in cognition (20), attention (21), and memory (22). Specifically, an increase in NREM sleep has been associated with improved long-term memory formation (23) and SOs have been shown to be independently associated with improved cognitive performance (24–28).

With respect to AD, sleep disturbance has been investigated as both a symptom of and a risk factor for the disease (4, 29, 30). Research has shown a correlation between sleep disruption and subjective cognitive decline, before MCI or AD manifest (31); less sleep fragmentation has been linked to lower risk for AD in older adults (32); and treating apnea-related sleep disturbance can delay the onset of MCI (33). Research relates sleep problems with AD pathology (Aβ and tau), showing a bidirectional relationship between sleep disruption and Aβ and tau accumulation in rodents and drosophila (30, 34–37). Consistently, increasing cortical Aβ accumulation is also associated with sleep fragmentation (34, 35).

Poor sleep correlates with Aβ and tau pathology severity among people with AD and MCI (38–42), and recent studies indicate associations between AD pathology and NREM sleep (40, 43). Recently, Fultz et al. (44) simultaneously measured functional magnetic resonance imaging (fMRI) studies measuring blood-oxygenation-level-dependent (BOLD) signals, electroencephalogram (EEG) and cerebrospinal fluid (CSF) and observed that at 0.05 Hz (SO that occurs during NREM sleep), there was a large-amplitude pulsatile flow of CSF.

Given that the glymphatic system has been shown to clear Aβ during sleep (36), along with the corollary that sleep disturbance permits the accumulation of Aβ, it is reasonable to conclude that techniques for improving sleep could be employed to counteract Aβ accumulation, and thus, memory decline and progression from MCI to AD.

## Gamma Band Oscillations and Brain Health

Gamma activity is composed of rhythmic oscillations that reflect underlying neural synchronizations. Although there is no agreed-upon frequency band that corresponds to gamma oscillations, the accepted lower and upper limits are usually in the range of 20–30 Hz and 80–120 Hz, respectively.

For the most part, gamma oscillations reflect the excitatory and inhibitory activity of interneurons. The cycle starts when excitatory neurons fire, triggering a synchronized discharge of inhibitory interneurons that impede the original excitatory neurons, briefly silencing them. The cycle restarts when the inhibitory signal wears off and allows the excitatory neurons to resume firing (45). Gamma oscillations can be observed throughout the cerebral cortex and correspond to the activation of the cerebral cortex. In the sensory cortex, gamma oscillations can be modulated by presence of sensory stimulation. For example, rhythmic visual stimuli at a certain frequency (i.e., flickering light) will elicit a brain response in synchrony with the frequency of those stimuli. In the visual cortex, exposure to visual stimuli (e.g., light bars) increases power in the 35–50 Hz range (46). In their study with cats, Gray and Singer (46) showed that the probability of neurons to fire in response to the presentation of optimally aligned light bars within their receptive field is greater when the stimulus has a peak frequency near 40 Hz. They also observed that this was a cortical response (i.e., a response of local neurons in the visual cortex) rather than a thalamic response.

Gamma-band oscillations have been associated with attention, working memory, and associative learning (47). During information-processing events, gamma oscillations allow for selective transmission of sensory information across distributed neurocircuits. Enhanced gamma activity is associated with the enhanced coherence between brain areas (48–51). For example, working memory processes have been associated with the coupling between the phase of theta (4–8 Hz) and the amplitude of gamma (52). In fact, Tort et al. (53) demonstrated that theta–gamma coupling strength directly correlates with increased performance during learning sessions in rats, suggesting theta-gamma coupling plays a role in memory recall (53).

Interestingly, human AD patients (54–56) and AD mouse models (57–59) show reduced power of oscillatory activity in the gamma range (30–100 Hz), which mediates essential neural functions including cortical arousal, sensory processing, working memory, attention-dependent stimuli, and higher order cognition (60–64). This deficit provides a valuable avenue to explore potential treatments for humans with AD or other cognitive impairments.

## Therapeutic Lighting Techniques

### Light for Entrainment and Phase Shifting of the Circadian System

It is well known that the light-dark cycle is the primary stimulus for synchronizing the circadian system, whose rhythms (e.g., the sleep-wake cycle) repeat approximately every 24 h. Ideally, light of the appropriate amount, spectrum, distribution, duration, and timing synchronizes the internal human circadian clock with solar day-night cycle to help maintain synchrony with work demand times (65). In humans, this synchronization occurs when the circadian system phase advances daily and outcome measures are generally downstream measures.

Lighting characteristics affecting the circadian system, as measured by acute melatonin suppression and phase shifting of dim light melatonin onset (DLMO), a marker of the timing of the biological clock, differ from those affecting our ability to read black font on a white paper. While less light than was originally demonstrated in the 1980s is needed to suppress nocturnal melatonin production, significantly higher amounts of light are needed to affect melatonin than those needed to activate the human visual system (66, 67). Indoor daytime workers, however, may spend their days in “biological darkness” because typical exposures to electrical lighting in indoor environments can be insufficient for entraining the circadian system (68). The lack of a strong light-dark stimulus to the circadian system can lead to sleep disturbances, such as those experienced by older adults, including AD patients, living in more controlled environments. Indeed, it has been shown that middle-aged adults receive ~58 min of bright light per day (69) while older adults in assisted-living facilities receive bright light for only 35 min per day (70). Adults in nursing homes see as little as 2 min per day.

Following the discovery of the intrinsically photosensitive retinal ganglion cell (ipRGC), a series of animal-model studies showed that circadian phase shifting can occur via input from the ipRGCs and/or the rods and cones, either alone or in combination (71, 72). These studies clearly suggest that, although the ipRGCs are instrumental to transduce the signal from the retina to the SCN (73, 74), melanopsin alone, the opsin that provides the ipRGCs with its intrinsic photosensitivity, is not needed for circadian phase shifting, and neither are rods and cones alone (74).

The peak spectral sensitivity for acute melatonin suppression and phase shifting of DLMO is close to 460 nanometers (nm) (75–77). Timing of exposure is also important for affecting the biological clock. The same stimulus presented in the morning—or after the minimum core body temperature, CBT_min_, that typically occurs in the second half of the night—will advance the timing of the clock in the following cycle (i.e., bedtimes and waketimes will be earlier the following day). Light given in the evening and early part of the night (before CBT_min_) will delay bedtimes and waketimes (78).

The duration of light exposure required for melatonin suppression depends on the light stimulus's magnitude (79). Continuous exposure to 74 μW cm^−2^ of a narrowband, short-wavelength light stimulus (peak close to 470 nm), for example, will elicit measurable melatonin suppression after 5–10 min. Continuous exposure to 2 μW cm^−2^ of the same blue light source, on the other hand, will elicit measurable melatonin suppression only after 90 min (79). Consistently, a 12-min exposure to 4,100 K fluorescent light (>6,000 lx at the cornea) more effectively phase-shifts circadian rhythms than exposures to lower light levels for longer durations (e.g., 6.4 h), as demonstrated by Chang et al. (80).

Finally, research shows that it is important to accurately measure light exposures over the 24-h day, as opposed to taking just a “snapshot” measurement of light exposure at one certain place and time (81, 82). Given that the circadian system appears to keep track of light exposure, knowing an individual's light exposure history over the past 24 h can help determine the best light prescription for the next 24 h (83). Therefore, a light treatment designed to promote earlier bedtimes should not be limited to reduced exposure to blue light in the morning but should instead control the total circadian light exposure during waking hours.

Amid ongoing investigation into the retinal mechanisms involved in photic stimulation of the circadian system, Rea et al. (77, 84) have proposed and continued to develop a model of human circadian phototransduction that is consistent with known retinal neuroanatomy and neurophysiology (85). The human circadian phototransduction model is based on the response of the ipRGCs (86), but it also includes responses from rods and cones, which have also been shown to provide input to the ipRGCs (71, 72). The ipRGCs, through the retinohypothalamic tract (RHT), transduce the combined photic signal to the master pacemaker, located in the suprachiasmatic nuclei (SCN). According to the Rea et al. model (77, 85), the cones provide indirect input to the SCN via synapses in the retina, one of which includes the spectrally opponent (blue vs. yellow) S-cone bipolar neurons that combine input from all three cone types to provide depolarizing-only (S-ON) input to the ipRGCs. The ipRGCs convey the combined photic information to the SCN. Following the model, while the intrinsically photosensitive response from the ipRGCs combines its signal with depolarizing “blue” response from the S-ON bipolar, the hyperpolarizing “yellow” response will not be received by the ipRGCs. In the case of light spectra that evoke a “yellow” response from the S-ON bipolar, the ipRGCs' response alone determines the photic information conveyed to the SCN.

### Continuous Light for Circadian Phase Shifting and Entrainment

Light therapy for improving sleep, mood, and behavior in AD patients has been the subject of investigation since the 1990s. A comprehensive review of the impact of light on circadian phase shifting and thus, entrainment has been published elsewhere (87), but a summary of some of these studies is presented below.

One of the first studies showing the positive impact of light on circadian rhythms of AD patients was published by Van Someren et al. (88). They studied the effects of increased levels of bright light during the day on 22 institutionalized older adults with severe ADRD. Ambient, unattended high light levels (>1,000 lux at the eye) delivered from ceiling luminaires and windows were used to deliver the intervention in spaces where most of the patients stayed during the day. The results showed that 4 weeks of bright light exposure over the course of a day improved disturbed circadian activity/rest rhythms in older adults with severe dementia.

Yamadera et al. (89) evaluated the effect of bright light exposure (3,000 lux for 2 h each morning) on cognitive functions and circadian rhythms in individuals with AD. The 27 participants experienced the intervention over four consecutive weeks. There was also a significant decrease in daytime napping, awakenings during sleep, and overall percentage of time of sleep increased for all participants. The authors speculated that the bright light exposure improved circadian rhythms and cognitive functions for participants in the early stages of the disease.

Not all of the studies, however, showed positive impacts of light therapy on sleep. Dowling et al. (90) investigated the effect of 1 h of bright light (≥2,500 lux) in the morning for 10 consecutive weeks on nighttime sleep, wake time during the day, and circadian rhythms in a study of 46 ADRD patients. No significant changes were observed in sleep efficiency, sleep time, wake time, or number of awakenings between the control group and the intervention group. However, the authors noted that the greatest improvements in the activity/rest rhythms occurred in those who experienced their 10 most active hours during typical hours of sleep at baseline.

In terms of long-term light therapy, Riemersma-van der Lek et al. (91) were the first to investigate the effect of long-term (maximum of 3.5 years) light exposure and oral melatonin in a study of 189 ADRD patients. Ceiling-mounted fixtures with fluorescent tubes were installed in a common living room. Results showed the light exposure attenuated cognitive decline, ameliorated depressive symptoms, and attenuated the increase in functional limitations by over half. Melatonin increased negative mood and withdrawn behavior, but shortened sleep latency and increased sleep duration.

Although studies to date have shown that light can be a powerful therapy for mitigating sleep disturbances and increasing memory consolidation in older adults with AD, not all of the studies showed consistent positive results (92, 93). This lack of consistency in the results is likely due to the fact that most of the studies to date lack a formal specification of the stimulus. The amount, spectrum, timing, distribution, and duration of the light exposures are not always described in the studies, and in many cases, not even taken into account when delivering the intervention in the field. In fact, a 2014 Cochrane review (94) of eight studies found insufficient evidence to recommend the use of light therapy for improving sleep and behavior in AD patients. The reviewed studies included different lighting interventions, however, and none of them controlled or measured the actual light dose that participants received during the interventions (94). The lack of control for the light delivery methods may have affected the outcomes of the analyses.

With the goal of addressing the issue of lack of control of the stimulus delivery and measurement, Figueiro and colleagues developed a tailored lighting intervention (TLI) designed to effectively affect circadian phase, and, thus, promote entrainment of the circadian system. Although circadian entrainment was not directly measured in the field, it was operationally defined that better circadian entrainment was associated with better sleep, mood and behavior. Figueiro and colleagues used the Rea et al. model to develop the TLI, which was used to deliver a robust light-dark pattern to persons with AD living at home (95) and in more controlled environments (96–98). In their studies, circadian-effective light was delivered from waking to 6 p.m. and circadian ineffective light was delivered during evening hours. Treatment duration varied from 4 weeks to 6 months. Results showed that the lighting intervention significantly reduced subjective sleep disturbances, increased objective sleep measures, an improved behavior, as observed by a reduction in depression and agitation scores (98). These findings were consistent with earlier studies showing that all-day or morning light exposures for short (4 weeks) and long (3.5 years) periods consolidated activity/rest patterns and improved subjective measures of depression (88, 91, 92, 99).

### Intermittent Light for Circadian Phase Shifting and Entrainment

In an experiment by Zeitzer et al. (100), 2-ms light pulses presented to the open eyes of six subjects every 1 min for a duration of 60 min during the early part of the night shifted (delayed) circadian phase by an average of 45 min, based on DLMO measurements. They also observed improvements in objective (electroencephalography) and subjective (Stanford Sleepiness Scale) measures of alertness. Although there was a significant phase shift of DLMO, the 2-ms light pulse intervention did not significantly reduce melatonin concentrations in the intervention night compared to the dark control night. While this was the first study showing the impact of pulsed light intervention in humans, its impact on animal circadian phase had been demonstrated before (101, 102).

At first, these findings appear to contradict predictions from models of human circadian phototransduction and the circadian pacemaker's response to light (103, 104), and suggest that melatonin suppression and phase shifting do not exhibit similar spectral and absolute sensitivities to light (67, 80, 105). Indeed, until the above-cited publication by Zeitzer et al. (100), most studies usually showed that prolonged exposure to light stimuli (i.e., longer than several milliseconds) would reliably phase shift DLMO and acutely suppress melatonin (79, 80).

The responses of the photoreceptor classes vary widely; cones respond very quickly to light stimuli (<50 ms) (106) while the ipRGC's response is much slower (>10 s) (86), and cones display a rapid, heightened response to pulses of bright light that is followed by a slow decay, even after the stimulus is extinguished (107, 108). One hypothesis tested by Figueiro et al. (82) was that, based on this slow decay of cone responses, flashes of bright, short-wavelength light could stimulate the SCN via the depolarizing, S-ON bipolar synapse without necessarily inducing a direct response from the ipRGCs. The Rea et al. model is therefore consistent with the hypothesis that a series of brief, short-wavelength light flashes stimulating the S-cones (a “blue” response) could convey photic information to the SCN. Conversely, a series of brief, longer wavelength green-, yellow-, or red-light flashes (i.e., a “yellow” response) would not elicit a response because it could not circumvent ipRGC sensitivity's high threshold and slow response.

**Figure 1:**
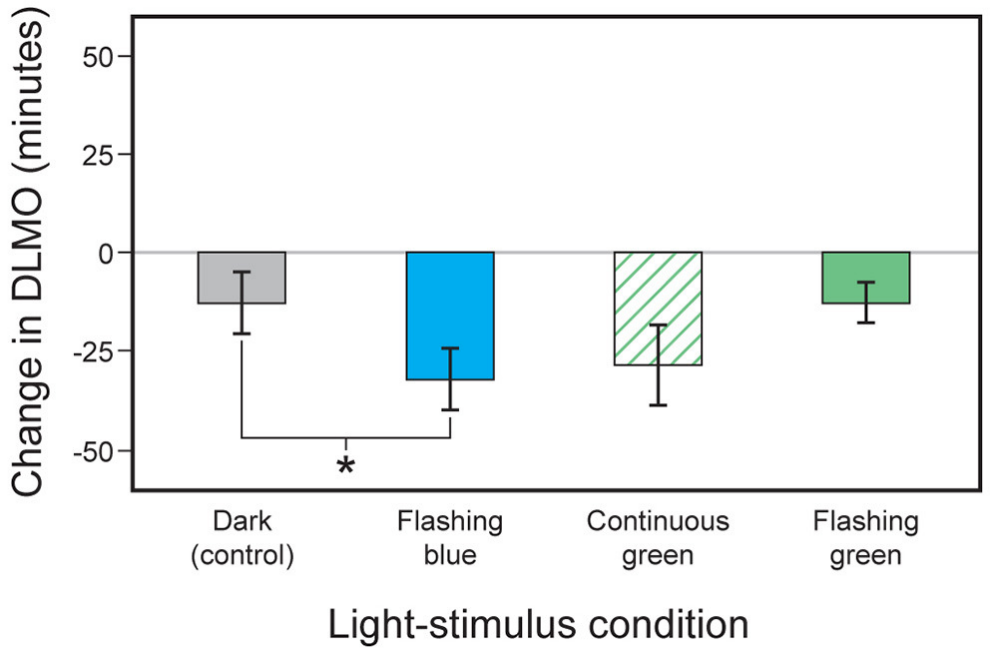
Changes in the timing of DLMO observed for the four light-stimulus conditions employed by Figueiro et al. (82). The error bars represent standard error of the mean. *p < 0.05.

First made possible by determining the human eyelid's spectral transmittance (109), subsequent laboratory research confirmed the efficacy of a light mask worn during sleep that delivered 60 min of continuous green (λ_max_ ≈ 527 nm) light through the closed eyelids of sleeping subjects for suppressing nocturnal melatonin and phase shifting DLMO (110). In a second publication, Figueiro et al. (82) tested the intermittent light hypothesis by comparing the effectiveness of flashing green and flashing blue lights on phase shifting of DLMO and on acute melatonin suppression in humans. They tested the hypothesis that brief pulses of short-wavelength light (blue) delivered in the early part of the night would delay DLMO and suppress nocturnal melatonin, while similarly delivered longer wavelength (green) light would not elicit the same circadian system responses. Three light-stimulus conditions were delivered to 16 subjects during sleep: (1) 111 W m^−2^ of blue (λ_max_ ≈ 480 nm) light presented as 2-s flashes at 1-min intervals for 1 h, (2) 131 W m^−2^ of green (λ_max_ ≈ 527 nm) light presented continuously for 1 h, and (3) the same green light presented as 2-s flashes at 1-min intervals for 1 h. After correcting for mean eyelid transmittance, the corneal irradiance levels of the flashing blue and the flashing and continuous green lights were set to values previously shown to be of approximately equal effectiveness for stimulating the human circadian system over a continuous exposure of 1 h (circadian stimulus or CS = 0.31 for blue and CS = 0.38 for green). Results showed that, compared to a dark control night and corrected for the natural drift of circadian phase, the flashing blue light evoked a statistically significant phase delay as measured by the incremental change in DLMO (Figure 1) and reliably suppressed melatonin. The flashing green light, conversely, did not reliably shift DLMO compared to the natural drift in phase observed for the dark control, nor did it reliably suppress nocturnal melatonin.

This study was followed by another study where the impact of the flashing blue lights was tested in a laboratory setting and a follow-up study conducted in the field, where exposures to light outside the intervention were measured using a calibrated personal device, but not controlled (111). In the laboratory study, they exposed 11 subjects to the flashing blue light delivered to close eyelids for 1 h in the early part of the night. The goal of this laboratory study was to determine the effectiveness of the light to suppress nocturnal melatonin. Melatonin levels during the intervention night, when the flashing lights were energized for 1 h, were significantly reduced compared to the control night, when the mask was worn but not energized. For the field portion of the study, 10 subjects completed a 2-week protocol. During the first week (baseline), they lived their normal lives while wearing light meters and actigraphs. At the end of the week, they came to the laboratory to collect saliva samples for DLMO measurements. During the second week (intervention), light masks delivering flashing blue lights were programmed to turn on 1 h after their bedtimes and remain energized for 2 h, turning off at least 1 h before predicted CBT_min_; therefore, the intervention was designed to delay their DLMO times. DLMO was significantly delayed (*p* = 0.003) after intervention compared to the control (average delay was 24.5 min), extending the results observed by Figueiro et al. (82) to field settings.

Following the laboratory and field studies confirming their hypothesis, Figueiro (112) tested the effectiveness of the flashing blue light on delaying the circadian system of those with early sleep onset living in their homes. They recruited 28 subjects (9 early awakening insomniacs) to participate in an 8-week, placebo-controlled, within subjects, crossover study. Two lighting conditions were tested, an active (flashing blue light delivered during early part of the night) and a control (flashing red light also delivered during the early part of the night). For each lighting condition, the subjects collected data during 2 baseline weeks and 1 intervention week. Saliva samples for DLMO measurements were collected at the end of each baseline and intervention week. Actigraphs and a calibrated light meter were used during the entire study.

After 1 week of the lighting intervention, results showed that exposure to the flashing (2-s pulses, every 30 s) blue light for durations ≤ 3 h, starting at least 1 h after bedtime, delayed DLMO by an average of 34 min. A control intervention, delivering a flashing red light (λ_max_ = 640 nm) exposure of the same timing and duration, however, delayed DLMO only minimally (6 min). Sleep start times were significantly delayed (by ~46 min) at day 7 compared to day 1 after the flashing blue light, and sleep efficiency was not affected by the intervention. Although DLMO and sleep start times were successfully delayed among subjects reporting a history of early awakening insomnia, it remained unknown whether the use of the light mask over longer periods would delay circadian phase and the timing of sleep among those with early sleep onset.

In order to investigate the effectiveness of the flashing light mask in a larger group in real-life conditions, using a crossover, placebo-controlled design, Figueiro et al. (113) exposed 32 subjects to either an active blue (λ_max_ = 480 nm) lighting intervention or a placebo red (λ_max_ = 640 nm) control through closed eyelids during sleep. The light stimulus was presented 1 h after bedtime for consecutive 8 weeks. The light was administered via custom-built light masks that delivered a series of 2-s light pulses at 30-s intervals for ≤ 2 h (~240 pulses/night). Subjective measures of sleep and depression (questionnaires) and objective measures of sleep (wrist actigraphy) served as dependent variables. Statistically significant (*p* < 0.05) improvement in seven of the eight subjective sleep measures were reported within both conditions, but no differences were observed between the two conditions. It should be noted that in this long-term study, subjects' daytime light exposures—particularly in the morning, which would have counteracted the effect of the flashing blue light in the evening—were not controlled. The authors hypothesized that if orange-tinted glasses had been worn by subjects in the morning, the impact of the intervention would have been more pronounced.

**Figure 2:**
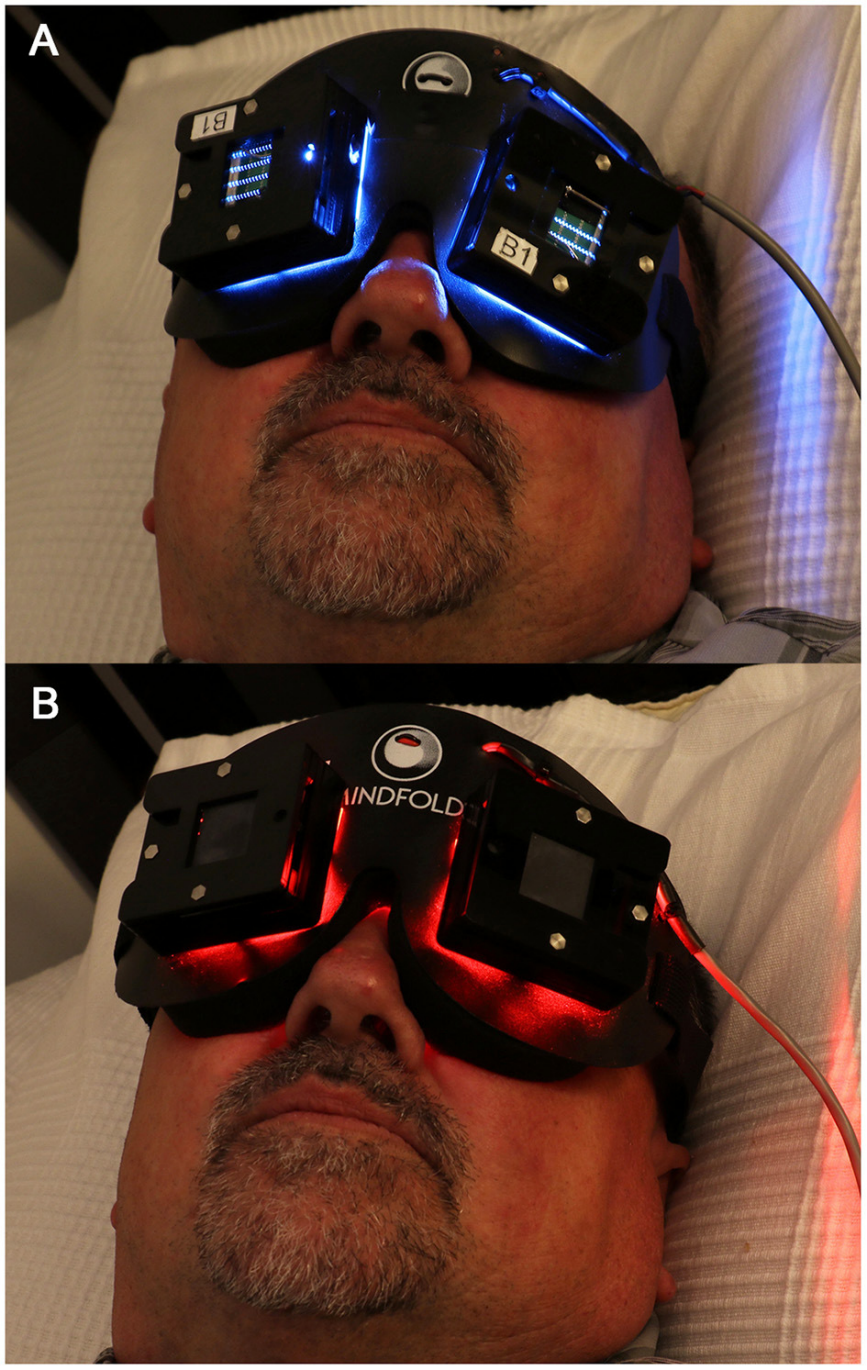
The light mask used to deliver light pulses through closed eyelids during sleep. The light mask contained 2 LED arrays for each eyelid: **(A)** active blue (λ_max_ = 480 nm, FWHM = 24 nm) or **(B)** placebo red (λ_max_ = 640 nm, FWHM = 25 nm). Written informed consent was obtained from the individual shown in this figure for the publication of this image.

This method of delivering flashing light through closed eyelids while people sleep (Figure 2) nonetheless holds considerable promise for the clinical correction of circadian misalignment, given that light applied close to the CBT_min_ has been shown to maximally shift the circadian pacemaker's timing (78, 114). The CBT_min_ occurs in the second half of the night, ~2 h prior to natural waking. For the success of this application, however, light exposures during wakefulness should be monitored at all times, perhaps aided by smartphone applications that provide timing recommendations for receiving and removing light stimuli. Future studies should examine how effectively such closed-loop systems might phase-shift the biological clock in real-life situations.

The effects of temporally modulated light stimuli that are delivered in the morning during wakefulness on circadian entrainment, rather than on circadian phase shifting, remains unexplored even though, as shown by the studies discussed above, the Rea et al. model (77, 85) permits qualitative predictions of the impact of temporally modulated light pulses for stimulating the human circadian system, as measured by acute melatonin suppression and shifting of the DLMO times.

### Intermittent Light at 40 Hz for Gamma Power Entrainment

Intermittent light stimuli permits gamma oscillations in the brain to resynchronize with the frequency of a flickering light; therefore, the administration of a visual stimulus flickering at 40 Hz will induce gamma oscillations at exactly 40 Hz (59). Such resynchronization has been shown to improve both learning and memory skills in a murine model (115). Because hippocampal gamma oscillations are linked to cognitive function, it is also assumed that these resynchronized oscillations are responsible for the increase in cognitive performance (116). The research in this area is still new and more work needs to be performed to determine the benefits of 40 Hz flashing lights on cognition.

Recent research has demonstrated that in hippocampal region CA1, as little as 1 h of optogenetic stimulation of parvalbumin (PV) interneurons, which are known to induce gamma oscillations (117, 118), could reduce Aβ peptides levels by ~50% in 5XFAD mice (59). A similar reduction was also observed in the visual cortex after mice were exposed to an external flickering light at 40 Hz. When 1-h stimulation was repeated daily over the course of 7 days, 40 Hz flickering light also reduced plaque pathology in the visual cortex of 6-month-old 5XFAD mice, and exposures of longer duration (i.e., 1 h per day for either 22 or 42 days) similarly reduced the loss of neuronal and synaptic density. The same stimuli also modified microglia morphology, consistent with increased phagocytic activity responsible for neuronal corpse removal in the brain (59, 119). Long-term (>6 weeks) daily exposure to 40 Hz flickering lights decreased microglia-mediated inflammation in P301S and CK-p25 mice, improved behavioral performance, and reduced loss of neurons in various parts of the brain (59, 120). This was confirmed by Garza et al. (121), who found that 40 Hz flickering light administered to healthy mice increased the expression of cytokines, which plays a central role in microglial recruitment. In a healthy brain, microglia exercise a protective function that restrains the accumulation of Aβ and may prevent neurodegeneration (122). It has been hypothesized that resynchronization of gamma oscillations stimulates recruitment of microglia (59, 123) which take part in ridding the central nervous system of undesirable features such as dysfunctional neurons or amyloid plaques (124). If proven to be viable, harnessing the maintenance role of microglia would be revolutionary, as it would permit the reduction of plaque accumulation using endogenous processes and thereby avoid the introduction of foreign substances to the body. It should be noted that the protective role of microglia in the brain may be lost in later stages of the disease. As toxic amyloid types accumulate in the brain, tau pathology builds up in stressed or damaged neurons, microglia transform into a destructive or inflammatory state that destroys synapses, secretes neurotoxic cytokines that harm neurons and may make matters worse, by spreading tau pathology (122).

It should also be stressed that this therapeutic approach appears to be effective for removing plaques via microglia activation/recruitment in murine models, but it remains unknown whether these findings will translate well to humans. If beneficial in humans, it will be important to determine at which stages of the disease this microglia activation by gamma entrainment switches from being beneficial to detrimental (122).

The first step to initiate this new line of research will be to show that 40 Hz flickering lights leads to an increase in gamma power in the brain. While we are a long way away from proving the efficacy of this intervention for improving cognition in those diagnosed with MCI and AD, a recent small pilot study by Sahin and Figueiro (125) demonstrated that 11 healthy young adults receiving a 40 Hz flickering red light stimulus induced a significant increase in 40 Hz power as well as an overall increase in low gamma power (30–55 Hz). Red light was used as the intervention stimulus because it does not affect the circadian system. There was also a significant correlation between the increase in 40 Hz power and a reduction in subjective sleepiness, as measured by the Karolinska Sleepiness Scale scores. The intervention did not have a significant impact on short-term performance and subjective sleepiness, as measured by the Karolinska Sleepiness Scale, compared to the dark control. According to authors, this may have been due to a “ceiling effect,” given that these were normal, healthy young adults. Moving forward, it will be useful to perform similar experiments in those with MCI for a longer period (weeks or months) to determine whether this increase in gamma power resulting from exposure to 40 Hz flickering light can improve cognition and delay transition to AD.

### Intermittent Light at 40 Hz: A Case for Dual Entrainment

In the only publication investigating the impact of an intermittent light at 40 Hz on both circadian clock genes and gamma entrainment, Yao et al. (123) compared mRNA levels of clock genes (BMAL1, Per2, and Clock) before and after exposure to 40 Hz flickering light using an AD mouse model (APP/PS1). They showed that 40 Hz flicker increased gamma in the visual cortex, decreased Aβ deposition, and decreased protein expressions of APP and phosphorylated tau in the hippocampus. They also showed that 40 Hz flicker lights partly restored CLOCK, BMAL1, and PER3 gene expression in the APP/PS1 mice, which was shown to be reduced compared to their controls. As noted by the authors, it was not possible from their studies to determine whether it was the light itself or the flicker that drove the observed changes. The wavelength of the light they used for their experiments, 462.8 nm (blue light), is close to the peak sensitivity of the circadian system, thus making it difficult to distinguish the cause of the expression level change. In order to confirm that the restoration of expression levels of genes was due to the flickering light, and not the short-wavelength light, it would be advised to conduct an identical experiment with either a random flicker or with a 40 Hz circadian-ineffective light. Ideally, these studies should be tested in humans and results will help develop new light therapies designed to promote both circadian and gamma entrainment.

## Discussion

Light-dark exposures reaching the retina are the major synchronizer of circadian rhythms to the local position on Earth. In general, this synchronization results from small phase advances that occur daily, give that the human circadian clock free runs with a period slight longer than 24 h. Therefore, to promote entrainment would be to advance the clock daily. In studies where light stimulus was carefully specified and measured, the effects on sleep, mood and behavior of various populations, in particular AD patients, were generally positive (95–97). For the most part, however, light therapy for circadian phase shifting resulting in better entrainment has been continuous, not intermittent. Only a few studies to date have investigated the effectiveness of intermittent light to phase shift the biological clock. This is perhaps due to the limited number of lab and field trials as well as to the fact that there is no clear justification for the use of intermittent light for promoting circadian phase shifting and entrainment alone.

One emerging line of research using phototherapy is the use of intermittent light delivering 40 Hz to promote entrainment of gamma oscillations in the brain. This line of research is still in its infancy and unlike with circadian entrainment, dose response curves to determine optimum light level, spectrum, timing and duration of exposure have not been developed for gamma entrainment therapy. The efficacy of light or sound alone and the combined use of light and sound therapy to promote gamma entrainment, while promising, is still under investigation (115, 120) and there are limited studies performed in human subjects.

Future research, and perhaps more-novel approaches, should investigate the development of a light therapy device that promotes phase shifting of the timing of the clock, and thus, entrainment of circadian rhythms while also promoting entrainment of gamma activity in humans. This would be a good justification for the use of intermittent light to promote circadian entrainment. For that purpose, the use of bright white light or narrow-band short-wavelength (blue) light would be needed, instead of the red light used in the Sahin and Figueiro (125) study. This dual entrainment therapy would likely have additive benefit because it has the potential to improve cognition directly, via entrainment of gamma waves, and indirectly, via improving circadian entrainment and sleep, which in itself is associated with better cognition. One issue that should be considered is the comfort and acceptance of the 40 Hz flickering light and its effectiveness when used in combination with ambient light. Moreover, although the 40 Hz flicker is outside the 0.1–30 Hz flicker range that is better known to induce epileptic seizures, at least one study (126) showed the onset of localized seizures at higher frequencies (60–100 Hz), and therefore, those who are known to suffer from epileptic seizures should not use the flickering lights. It has been suggested that AD patients are more prone to seizures (127), and it is not known whether the flickering lights would have a different effect in those with neurodegenerative diseases compared to healthy people. Nevertheless, intermittent flickering light therapy shows a lot of promise due to its affordable price and easy administration. Initially, testing these interventions in MCI and mild AD patients would likely be the most beneficial, given that this population suffers from *both* sleep disturbances and cognitive deficit.

In summary, this dual entrainment intervention may be very beneficial to those with neurodegenerative diseases. If the same lighting device can be used to target both, gamma and circadian entrainment, this could be easily incorporated in the homes of those with early stages of neurodegeneration. While the impact of light for promoting entrainment in those with early and late stages of AD has been shown in the field (95–97), it is yet to the determined whether the flickering light will also have a positive effect in those who are in early, and more importantly, at later stages of the disease. This is a new area of research, but given that preliminary evidence shows its potential as an important therapy for those suffering from sleep disturbances or cognitive impairment, new research should investigate the additive effect of these two therapies.

## Author Contributions

MF conceptualized and wrote the manuscript. SL wrote parts of the manuscript. Both authors reviewed and approved the final version.

## Conflict of Interest

The authors declare that the research was conducted in the absence of any commercial or financial relationships that could be construed as a potential conflict of interest.
